# Impact of etravirine use on hospitalization rates among highly pre-treated failing HIV-1 infected individuals between 2005 and 2011

**DOI:** 10.7448/IAS.17.4.19781

**Published:** 2014-11-02

**Authors:** Jean-Marc Lacombe, Cecile Goujard, Marc-Antoine Valantin, Arnaud Cheret, Rima Lahoulou, Pierre-Marie Girard, Dominique Costagliola

**Affiliations:** 1 INSERM and Sorbonne Universités, UPMC Univ Paris 06, Pierre Louis Institute, Paris, France; 2 AP-HP, Hôpital Bicêtre, Service de médecine Interne, Le Kremlin Bicêtre, France; 3 AP-HP, Hôpital Pitié Salpétrière, Service de Maladies infectieuses et tropicales, Paris, France; 4 JANSSEN, Issy-les-Moulineaux, France; 5 AP-HP, Hôpital Saint-Antoine, Service des maladies infectieuses et tropicales, Paris, France

## Abstract

**Introduction:**

Etravirine (ETR), a non-nucleoside reverse transcriptase inhibitor available in France since 2006, is indicated for the treatment of HIV-1 infection in combination with a ritonavir boosted protease inhibitor (PI) in antiretroviral treatment-experienced adult patients. To assess its impact in routine clinical care, our objective was to compare hospitalization rates in highly pre-treated failing HIV-1 infected individuals between 2005 and 2011 depending on whether or not they received ETR+PI.

**Methods:**

From the French Hospital Database on HIV (ANRS CO4), we selected highly pre-treated individuals (prior exposure to at least 2NRTI, 2PI and 1 NNRTI) with a viral load (VL)>50 copies/mL initiating a new regimen between 2005 and 2011. Hospitalization rates were calculated for each calendar month and depending on whether patients never received ETR+PI at any time or during months before initiating ETR+PI (no ETR+PI) or during months after initiating ETR+PI (ETR+PI), using an intention to continue treatment approach. Poisson regression models were used to compare incidence between the two groups, after adjustment for potential confounders (age, transmission group, origin, AIDS, PCP prophylaxis, viral load, CD4, nb of previous ARV).

**Results:**

Overall 3884 patients fulfilled inclusion criteria. Among them 838 (21.6%) received ETR+PI at least once. Among all enrolled patients, 35.8% had CD4 <200/mm^3^, 17.5% had VL >100,000 copies/ml, 42.8% had had an AIDS event and 47.8% had received more than 10 different antiretroviral drugs. There were 2484 hospitalizations in 808 individuals over 13,986 patient-years. The hospitalization rates for 1000 P-Y were 169.0 for the ETR+PI group and 179.3 for the no ETR+PI group. After adjustment, the corresponding figures were 144.8 for 1000 P-Y and 192.7 for 1000 P-Y respectively, with a relative risk estimated as 0.75 (95% CI 0.67–0.84).

**Conclusions:**

Access to ETR+PI between 2005 and 2011 was associated with a 25% reduction in the hospitalization rate among highly pre-treated failing HIV-1 infected individuals.

